# Current Challenges in the Development of Platelet-Rich Plasma-Based Therapies

**DOI:** 10.1155/2024/6444120

**Published:** 2024-08-09

**Authors:** Jon Mercader-Ruiz, Maider Beitia, Diego Delgado, Pello Sánchez, Begoña Porras, Irene Gimeno, Sergio González, Fernando Benito-Lopez, Lourdes Basabe-Desmonts, Mikel Sánchez

**Affiliations:** ^1^ Microfluidics Cluster UPV/EHU BIOMICs Microfluidics Group Lascaray Research Center University of the Basque Country UPV/EHU 01006, Vitoria-Gasteiz, Spain; ^2^ Advance Biological Therapy Unit Hospital Vithas Vitoria 01008, Vitoria-Gasteiz, Spain; ^3^ Arthroscopic Surgery Unit Hospital Vithas Vitoria 01008, Vitoria-Gasteiz, Spain; ^4^ Microfluidics Cluster UPV/EHU Analytical Microsystems & Materials for Lab-on-a-Chip (AMMa-LOAC) Group Analytical Chemistry Department University of the Basque Country UPV/EHU 48940, Leioa, Spain; ^5^ Basque Foundation of Science IKERBASQUE 48009, Bilbao, Spain

## Abstract

Nowadays, biological therapies are booming and more of these formulations are coming to the market. Platelet-rich plasma, or PRP, is one of the most widely used biological therapies due to its ease of obtention and autologous character. Most of the techniques to obtain PRP are focusing on new processes and methods of optimization. However, not enough consideration is being given to modify the molecular components of PRP to generate more effective formulations with the aim of improving PRP treatments. Therefore, this review covers different novel PRP-obtaining methods that attempt to modify the molecular composition of the plasma.

## 1. Introduction to Biological Therapies

Biological therapies refer to any type of medical therapy that is derived from living organisms such as humans, animals, or microorganisms. This contrasts with traditional nonbiologic pharmaceutical drugs, which are synthesized in a laboratory via chemical processes [1]. The first biological therapy on the market appeared in the 19^th^ century with the discovery of the vaccines [2] and the insulin [3]. Since the 1990s, this number has vastly multiplied being now available to treat a wide variety of medical conditions such as cancer and autoimmune diseases [4].  Table 1 shows different classifications of biological therapies employed in the current times. Although these therapies are promising, they all come with different benefits and potential risks [1, 16].

Over the last decade, biological products within the framework of regenerative medicine became promising tools in the therapeutic field of different medical specialties such as dentistry, traumatology, sports medicine, aesthetic medicine, or even in different types of surgery as tools to promote tissue repair, among others [17–20].

The use of these treatments represents a paradigm shift in the way medicine is dealing with injuries and pathologies suffered by the patient: the aim is not to eliminate or replace the affected tissue but to take advantage of biological processes to stimulate tissue repair. Moreover, these techniques also seek to solve the problem from its source, and not only to alleviate the symptoms as is the case of analgesic pharmacology [21]. The versatility of biological products allows to use both conservatively and as an adjuvant to surgical interventions to improve results [22–24]. However, a good knowledge of the technique used in the treatments is necessary to achieve optimal clinical efficacy. It is essential to be clear about the type of product being used, as well as its characteristics, in order to apply it to the right patient and indication, and in the right way [25]. The main biological products for tissue repair can be grouped into cellular products and hemoderivative products, being platelet-rich plasma (PRP), a hemoderivative product, the most used one as cell therapies have certain limitations [26, 27]. In fact, current regulatory agencies tend to include cell therapies within the group of the so-called *Advanced Therapies*, which have a very restricted use, which is only permitted in certain situations such as clinical research or for compassionate use. For all these reasons, the application of cell therapies is still very limited [28] and their scientific and clinical background is scarcer than that of PRP, which has been widely used in recent years [29].

## 2. PRP

PRP is an autologous biological hemoderivative product within the framework of regenerative medicine, which has gained great value in the last few years due to its easy preparation and low-cost processing. It is applied for the treatment of different pathologies, promoting tissue regeneration and reparation [30]. Its basic principle is to obtain a fraction of blood plasma containing platelets in a higher concentration than that at baseline blood levels [31], according to the Spanish Medicines Agency [32]. From a pharmacological point of view, it is very difficult to define [33], as PRP has a large number and variety of active substances, often even antagonistic. Indeed, although PRP contains anabolic growth factors (GFs), it also contains many biomolecules that may antagonize its restorative effect on tissue metabolism [34, 35]. PRP therapeutic potential relies on the synergistic action of the biomolecules present in both plasma and platelets, and their content, which is the central core of this therapy [36, 37].

### 2.1. Platelets

**Figure 1:**
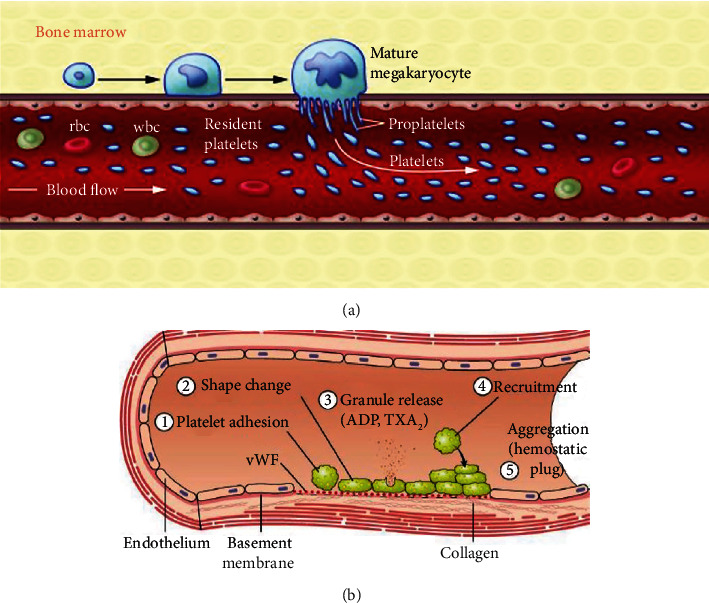
Graphical representation of platelet formation and its role in hemostasis. (a) After the maturation of the megakaryocyte in the bone marrow, it arrives to the endothelial barrier and releases its prolongations forming the platelets [41]. (b) Platelets, after different signaling pathways, are accumulated in the damaged area creating a plug that participates in the repair of the tissue [42].

Platelets are anucleate subcellular fragments with a life span of 7–10 days and present in blood at a concentration of 150,000–450,000 platelets *μ*L^−1^. Structurally speaking, platelets have an irregular disc shape of 2–5 *μ*m in diameter and 0.5 *μ*m of thickness [31, 38]. Megakaryocytes of the bone marrow are the precursors of platelets [39], which, after maturation, migrate to the endothelial barrier releasing the precursors to the bloodstream, also known as thrombocytes [40] (Figure 1(a)). Platelets are continuously moving toward the edge of blood vessels, being essential to recognize endothelial injuries [38].

Platelets contain a membrane with a large network of receptors that trigger key intracellular signals for the development of their functions [43]. Upon vascular injury, the subendothelium is exposed promoting von Willebrand factor (vWF) to deposit onto the exposed collagen. Platelets get attached at the site of the injury via interactions with these adhesive extracellular molecules. Once adhered, activated platelets aggregate and interact with the polymerizing fibrin network, tissue, and other platelets to form a haemostatic plug, which participates in tissue homeostasis and repair [44] (Figure 1(a)).

As mentioned above, the therapeutic potential of PRP is based on achieving a plasma fraction with a platelet concentration higher than blood levels. Even if there is still controversy to define an optimal platelet concentration, many studies consider a 1.5–3-fold increase in the concentration of platelets, the most favorable ones [31, 34, 45]. Indeed, other studies say that a higher platelet fold could reverse the therapeutical effects of PRP [46].

### 2.2. Molecular Content

The internal contents of platelets are stored in different small secretory granules called *α*-granules, *δ*-granules (dense bodies), and *λ*-granules (lysosomal type organelle) [47]. The material present in these granules is synthesized by the original megakaryocyte, as well as captured by platelets through endocytosis. The *α*-granules are the most abundant granules and have the highest content of active biomolecules related to tissue repair. Indeed, they constitute 10% of platelet volume over a number of 50–60 *α*-granules per platelet [48]. Hundreds of molecules including membrane bound and soluble proteins have been identified inside these organelles, including adhesive proteins, fibrinolytic and coagulation factors, antimicrobial molecules, cytokines, and GFs, among others [48]. Essentially, the interaction between these GFs and the surface receptors of the target cells activates the intracellular signalling pathways [49]. Adult mesenchymal stem cells, fibroblasts, osteoblasts, epidermal, and endothelial cells express cell membrane receptors, which are specific to each GF [50, 51]. It is therefore suggested that the GFs activate several cell types involved in tissue healing and thus promote healing of soft tissue and regeneration of bone [20]. When platelets activate, it occurs the release of molecules and other elements such as platelet microparticles or exosomes, which have a high antifibrotic and anti-inflammatory immunomodulatory effect [52]. Exosomes are small vesicles of 100–400 nm that are involved in cell communication and transport various proteins as well as other biomolecules such as genetic material [38, 53–55].

GFs include the platelet-derived GF (PDGF), which is a potent chemotactic factor for several cell types, having an important effect on tissue repair. Another growth factor with a strong presence in platelets is the transforming GF beta (TGF-*β*), whose effects are diverse and can be of different nature depending on the molecules and cells with which it interacts.

It influences early tissue repair responses, mesenchymal stem cell differentiation processes, and the maintaining of cartilage and subchondral bone. Other regulatory factors for tissue repair are the vascular endothelial GF (VEGF), the epidermal GF (EGF), or the fibroblast GF (FGF), which play key roles in cell migration, proliferation, differentiation, or in angiogenesis. In addition, extraplatelet molecules such as the insulin-like GF-1 (IGF-1) or the hepatocyte GF (HGF) also play a crucial role in the tissue repairing process; they are GFs that enhance regenerative processes as well as the modulation of inflammatory processes. The prominent GFs and their biological functions are detailed in Table 2 [56–63].

PRP is therefore a cocktail of thousands of biomolecules coming from plasma and platelets that regulates haemostasis, coagulation, tissue repair and regeneration, inflammation, cell behaviour, and defence against microorganisms, among other biological processes. All this therapeutic potential depends to a large extent on their composition, which can vary according to the method of obtaining [31] the PRP and the activation process [64].

### 2.3. Preparation of the PRP

PRP can be formulated in multiple ways with no consensus on a protocol that could be internationally used to standardize and facilitate their formulation [33]. The basis of the preparation method relies on the concept of differential centrifugation protocols [65]. Each component of the whole blood has a different density and separates into distinct layers when they are spun when using a centrifuge [66]. These layers consist of a lower fraction of erythrocytes, a thin layer of leukocytes or *buffy coat*, and, finally, a plasma fraction with platelets at certain concentration depending on the used centrifugation protocol [31].

**Figure 2:**
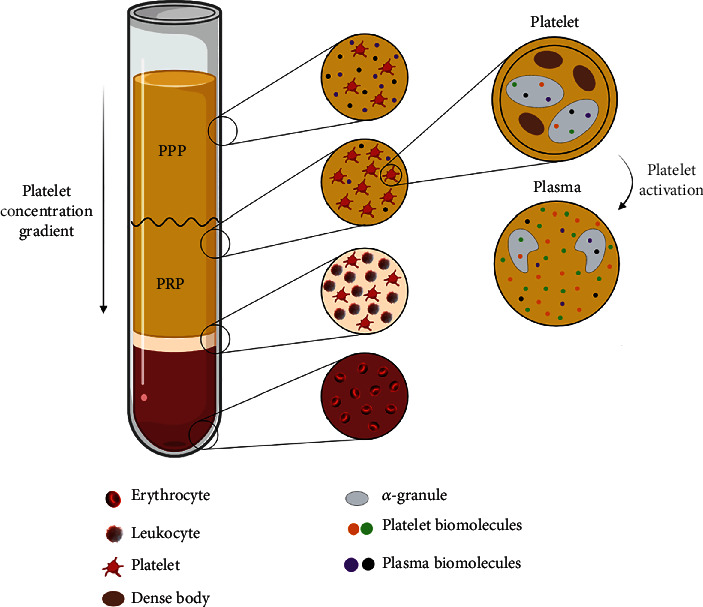
Graphical representation of a PRP-obtaining method in which a platelet gradient is achieved. Whole blood is separated into three fractions: one full of erythrocytes, another white-colored fraction called *buffy coat* formed by platelets and leukocytes, and finally, the plasma area. PRP area represents 2 mL of the total plasma fraction and contains two to three times more platelets than the PPP fraction. After activation of the platelets, all the biomolecules from *α*-granules are released into the plasma [31].

Centrifugations can be single or double, with a centrifugal force of between 350 and 2000 × g and a centrifugation time of 3–15 min [65]. Depending on the parameters used in these centrifugations, the number of platelets may vary. In fact, plasma fractions with lower or higher platelet concentrations can be obtained, or even fractions with platelet concentration gradients. A PRP preparation protocol example is shown in Figure 2, where a platelet concentration gradient is achieved along the plasma fraction and the platelet poor plasma (PPP) fraction contains platelets below 50 × 10^6^ platelet mL^−1^ levels [67].

### 2.4. PRP Types and Classification

As previously mentioned, PRP is a plasma fraction with a high concentration of platelets. Moreover, PRP can be modified by adding other elements to its formulation to treat specific pathologies [68]. Therefore, there is a wide range of PRP formulations depending on its use.

The main variables that can condition PRP are the number of platelets [69–71], the presence or absence of erythrocytes and leukocytes [72], the use of PRP in liquid or gel format [73], and, related to this, the way of activating the platelets [64]. This generates many different products under the term PRP that need to be well classified due to a lack of standardization [74]. In response to this, several classification systems have been proposed to classify these therapies. The most recent system was proposed by Kon et al. [75], which clearly and simply set out a classification system for PRP. The code is a sequence of six digits grouped in pairs indicating parameters of platelet composition, purity, and activation with the aim of unifying the way that PRP is classified for comparison and a more precise definition of the product.

#### 2.4.1. Platelet Concentration

It is important to note that although PRP can be formulated with different platelet concentrations, a higher number of platelets is not strictly related to a better effect [70].

PPP is the blood fraction with less concentration of platelets comparing with PRP, and it does not have the same therapeutic advantages due to the reduction in platelet content, GFs, and cytokines [34]. Although plasma fraction contains the proteins responsible for the coagulation cascade, according to some studies, PPP is not as effective as PRP [76–78]. Indeed, the effect of platelet concentration was demonstrated by several studies. A platelet ratio below 1.5 times compared to baseline levels showed no promotion of bone regeneration, whereas levels between 6 and 11 times higher than baseline revealed an inhibitory response in bone regeneration [79]. However, a platelet ratio of 1.5–6 times compared to basal levels has been proven to be effective in numerous treatments [80–82], with the 1.5–3-fold concentration being the most favorable in most pathologies [83, 84].

#### 2.4.2. Presence of Erythrocytes

Regarding to the presence of erythrocytes in the PRP, the activation of platelets in the presence of red blood cells (RBCs) leads to its own degradation processes, such as hemolysis and erythrocytosis, generating products that promote inflammation, cellular stress, or even cellular death [72, 83], which would hinder the beneficial action of PRP. In addition, Gersh, Nagaswami, and Weisel saw that presence of RBCs in PRP triggered variability in the fibrin network structure, individual fiber characteristics, and overall clot viscoelasticity compared to the absence of these cell type [85].

#### 2.4.3. Leukocyte Concentration

When separating plasma from the rest of the blood fractions, leukocyte or white blood cell (WBCs) layer could be collected. The leukocyte-rich PRP (LR-PRP) includes leukocytes in the autologous plasma fraction while the leukocyte-poor PRP (LP-PRP) excludes them [34]. The foremost question between these two types of formulations is whether having WBCs helps or affects the treatment response. Some studies said that the content of leukocytes might play a key role in the antimicrobial response and thus enhance the release of GFs. WBCs also secrete some proteinases that play an important role during the wound healing process [53, 86]. In contrast, other authors stated that the use of leukocytes inhibits the wound healing process by the release of reactive oxygen species (ROS) by neutrophils in the affected tissue area [51]. In fact, the presence of WBCs induces high levels of proinflammatory cytokines that may activate the inflammatory and catabolic changes that counter the beneficial effect of GFs in tissue repairing [87]. To be more specific, it was found that leukocytes activate the transcription factor NF-*κ*B pathway [88]. This factor regulates multiple aspects of innate and adaptive immune functions and serves as a crucial mediator of inflammatory responses. Moreover, it induces the expression of proinflammatory genes, including those encoding cytokines and chemokines, being its regulation crucial to prevent inflammation [89].

Overall, there is a lack of adequate studies on the potential positive or negative effects of leukocytes in PRP formulations, so the optimal concentration of WBCs is not clarified yet.

#### 2.4.4. Activation Methods for Platelets

The optional activation of PRP is the last part of the process of PRP treatment. During platelet activation, platelets release their contents but also trigger the polymerization of fibrinogen into a fibrin mesh [90] keeping biomolecules on it [91], resulting in a controlled release of those molecules while the fibrin mesh is degraded [17, 31].

Activation of platelets can be endogenous or through the addition of an exogenous clotting factor, producing different effects which influence the kinetics of GFs [64, 92].

Exogenous activation involves physical methods, such as freeze-thaw cycles, or by the addition of certain substances such as calcium chloride or human/bovine thrombin [92]. The main difference between the addition of CaCl_2_ and thrombin is that the last causes rapid aggregation and activation of platelets which leads to a decrease in the number of GFs. Regarding human or bovine thrombin, although both achieve hemostasis at the same time, bovine thrombin demonstrated seroconversion for some antibodies assayed than patients who received human thrombin [93], being the human thrombin the gold standard for platelet activation. Besides, some GFs have a short half-life and might degrade in minutes to hours if they are not immediately applied in treatment [94, 95]. On the other hand, CaCl_2_ generates a less condensed fibrin matrix, allowing a slower release of GFs over a period of 7 days, enhancing cell migration and wound healing [58]. What is more, it avoids the use of exogenous biological elements such as thrombin and the local hypocalcemia that can be caused by calcium chelating anticoagulants used previously in the blood collection to prepare the PRP. The advantage obtained by combining both agents is the benefit of creating an improved autologous gel or scaffold matrix that could be used in surgical interventions to fill several wound injuries maintaining a gradual GF release [31].

Regarding the endogenous activation, some methods propose the administration of nonactivated PRP as platelets are physiologically activated once they enter the body by means of endogenous factors such as calcium, collagen, thrombin, ADP, fibrinogen, vWf, or serotonin, among others [96].

#### 2.4.5. Gel State Formulation

Gel formulations provide the damaged tissue with a fibrin matrix that acts as a structural support during the tissue repair process [97]. PRP gels are produced by the addition of thrombin or calcium chloride to the PRP fraction activating platelets and triggering the coagulation cascade, consequently forming a fibrin matrix, which enables the controlled delivery of GFs [34, 98].

### 2.5. PRP Applications and Efficacy

The use of PRP has increased considerably in recent years due to the effectiveness of this treatment in different medical areas. Dermatology is one of the fields in which PRP has been effectively applied [19, 99, 100], achieving promising results in different areas such as facial rejuvenation, scar and wrinkle reduction, and its use in the hair with the aim of reversing alopecia, among other treatments. The field of PRP in dermatology has only been exploited for a few years. Therefore, more clinical studies are needed to demonstrate a significant improvement over other treatments and its use in new treatments for different pathologies [19].

Over the last few years, the use of PRP has been increased in the field of oral and maxillofacial surgery with the purpose of achieving bone regeneration, a rapid wound healing, and a reduction of bleeding after surgery [101]. During the first years, studies showed that the use of this therapy was beneficial for periodontal regeneration and regeneration of bone around the bone grafts. Nevertheless, future research in this field should be directed toward the implementation of well-designed, adequately powered clinical trials. The results of such trials will help elucidate the role of PRP in periodontal and other oral surgical settings [102, 103].

The area of traumatology and orthopedic surgery has generated much interest in the use of PRP for the treatment of certain pathologies [17, 20]. One of the applications is the regeneration of bone tissue. Clinical and experimental studies about the osteogenic effect of PRP have shown that GFs enhance gene expression of Type 1 collagen, osteocalcin, and osteopontin, thus promoting new bone formation [104]. Although, more studies are needed, according to a study, it has been reported that the use of PRP together with bone grafts resulted in a greater bone density [105].

Tendon and ligament healing were one of the first investigations performed with the PRP. Tendinopathy happens by natural aging and by repetitive stress. The injured tendons heal with scar tissue, and that adversely affects its function. Some studies have demonstrated good results by showing improved tenocyte proliferation and collagen formation when PRP was applied [104]. Regarding ligaments, in studies conducted on sports, the evidence show improved time of healing, reduced pain, and reduced time to return to sport [106].

In terms of cartilage damage, PRP induced a high mitogenic response in the chondrocytes enhancing the number of collagen-producing cells and increasing cell apoptosis. All this led to a high expression and synthesis of collagen by chondrocytes [104]. Related to this, osteoarthritis (OA) is a chronic degenerative disease of articular cartilage and other joint tissues that affects joint functionality and where PRP injections have shown positive results [107, 108].

Finally, among the therapeutic alternatives to address peripheral nerve injuries, nerve GF (NGF), brain-derived neurotrophic factor (BDNF), and PDGF are some of the components of PRP that can enhance nerve regeneration. The biomolecules present in PRP are instrumental agents that act as key drivers of full functional nerve recovery, promoting processes such as neuroprotection, modulation of inflammation, angiogenesis, enhancement of axonal growth, and prevention of denervated muscle atrophy [109].

Theoretically, the use of PRP in orthopedic treatments has important implications for accelerating and supporting the healing process of the musculoskeletal injuries [110–112]. The versatility of PRP-based products makes them an optimal biomaterial to be applied to the injured area by means of liquid infiltrations, reducing pain and improving the functionality of the joint in degenerative pathologies. Moreover, PRP can also be applied in solid form as a membrane during surgery [113]. Thus, the use of PRP in osteochondral focal defects as “scaffolds” for GFs also generates a proliferative, migratory, and chondrogenic environment of endogenous mesenchymal progenitor cells that favors the reparative environment [114]. The cellular effects generated as a consequence of the application of PRP to the injured tissue are intended to achieve a clinical result that favors and improves the recovery of the patients.

## 3. Recent Methods to Obtain PRP Focused on Platelet Concentration

Despite the expansion of PRP applications in recent years, and the large number of methods for its obtention available on the market, they are all based on the principle of using different centrifugation programs to separate blood components [33]. By varying the number of centrifugations and certain parameters, higher or lower platelet and WBC counts can be obtained.

Centrifugation is a standard method that has been used for a long time, and it works well to obtain PRP, but it has its limitations. Depending on the number of centrifugations, the samples require more or less manipulation, increasing the waiting time and the risk of platelets settling to the bottom of the tube [115, 116]. In addition, depending on the centrifugation force, the platelets are subjected to higher forces that may lead to their activation [117].

Centrifuge-based systems have several disadvantages—they required user intervention and do not concentrate all the biomolecules—and it is difficult to control the concentration yield [118, 119].

In order to optimise the process, new methods of obtaining PRP that do not involve centrifugation have been developed in recent years. For this purpose, different techniques have been used depending on the volume of PRP to be obtained and its applications, thus creating two types of system: macroscale and microscale.

### 3.1. Macroscale Systems

Macroscale systems involve any system capable of obtaining volumes of PRP of 1 mL or higher. Jacofsky et al. [120] developed a single use, disposable platelet concentration device based on a filtration system. They compared the performance of their device with a centrifuge-based PRP system. To obtain PRP, they transferred two 55 mL samples of whole blood from donors to a 60 mL syringe with 5 mL of citrate dextrose anticoagulant. One of the syringes was centrifuged to obtain 7 mL of PRP, and to the other one, they applied the filtration system to obtain the same volume. The filtration process consisted of three phases: first, the contents of the syringe were mixed with an aqueous solution and passed through a blood filter to capture the platelets. Afterwards, another aqueous solution was passed through the filter to release and reincorporate the platelets, resulting in a final volume of 7 mL. The results showed that the separation and concentration process take 13.6 ± 0.1 min using the centrifuge technique, and 8 ± 2 min using the filtered method, making the latter 40% faster than the former. In addition, the results showed a similar number of concentrated platelets and GF concentrations when comparing the two systems. In this research, the blood was diluted with an aqueous solution modifying the initial composition of the plasma, altering its autologous nature. However, no test was performed to find out if the platelets were activated during the filtration process.

**Figure 3:**
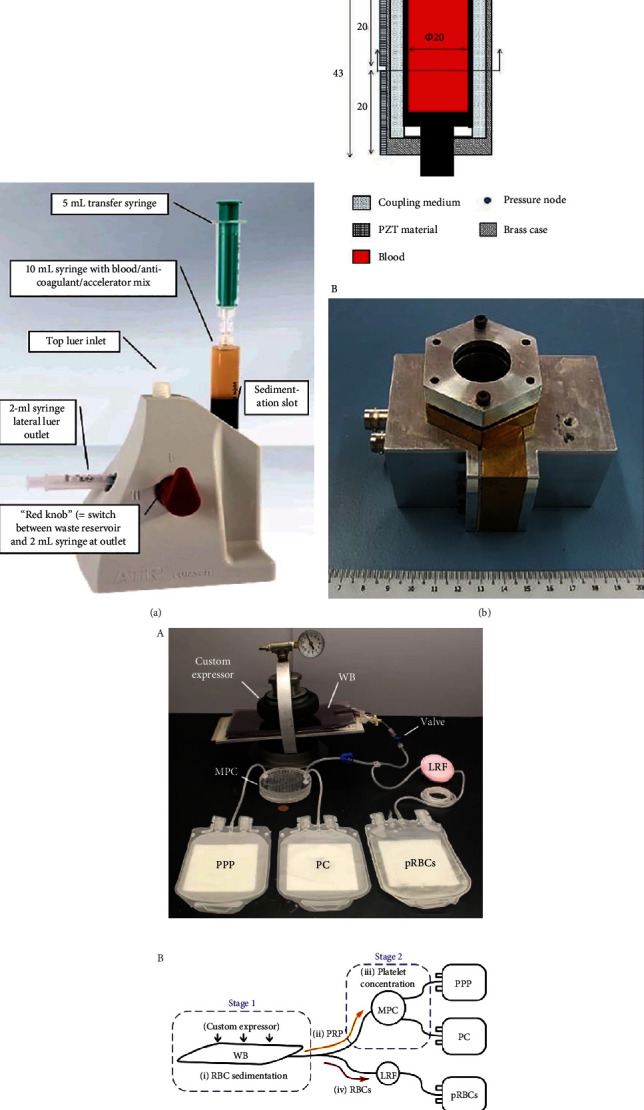
Methods to obtain high volumes of PRP. (a) Representation of the sedimentation system. (b) Ultrasound device to obtain PRP. (A) Section of the device where the blood is placed and submitted to ultrasonic waves. (B) Device used for ultrasonic PRP separation. (c) Portable system to obtain PRP. (A) General set up for the separation of plasma and concentration of platelets. (B) Stages needed to obtain different plasma formulations. Whole blood (WB), microfluidic platelet concentrator (MPC), leukoreduction filter (LRF), platelet poor plasma (PPP), platelet concentrate (PC), and processed units of packed red blood cells (pRBCs).

Schmolz et al. [121] created another system for the obtention of PRP within 2–3 h (Figure 3(a)). They used a special device involving separation systems: sedimentation and filtration. For the preparation of the PRP, they collected 8 mL of whole blood into a 10 mL syringe with an anticoagulant and a sedimentation accelerator. The syringe was placed in a special device and was left there for about 60 min to promote sedimentation. Next, the supernatant was moved from the syringe into a second one, followed by which 3.5 mL was injected into a specific inlet of the device, passing the plasma through a membrane filter where the platelets become trapped. Then, another solution was passed through the membrane, acting as a washing buffer to remove the anticoagulant and the sedimentation accelerator. Finally, another solution was passed twice through the same membrane, to promote the elution of the platelets' GFs for the obtention of the PRP solution. This centrifugation-free study reported no information about platelet concentration, but they demonstrated that there were substantial quantities of GFs, TGF-*β*, and PDGF. Furthermore, the concentration of other important GFs were higher than that found in the plasma before obtaining the PRP. Conversely, although this system directly releases the content of the platelets, the time needed for the PRP to be obtained and the numerous steps in the procedure could present a disadvantage. Furthermore, as in other methods, the autologous nature of the formulation was altered due to the use of buffers during the PRP obtaining process.

Wu et al. [122] developed an ultrasound device to obtain PRP by creating standing waves using piezoelectric ceramics inside a syringe. The syringe was filled with 10 mL of whole blood and, subsequently, RBCs accumulated at certain locations of pressure nodes due to the acoustic radiation force (Figure 3(b) (A)). The cell cluster that formed had a high sedimentation force, thus separating blood components into different fragments, providing 4 mL of PRP within 10 min. This research group demonstrated that the use of an ultrasonic device (Figure 3(b) (B)) displayed advantages over that of a commercial centrifugal double syringe, achieving a higher platelet recovery rate (%) of 79 ± 9 (versus 54 ± 10), an RBC removal rate (%) of 99.0 ± 0.1 (versus 78 ± 10), and a platelet-fold increase of 2.1 ± 0.1 (versus 1.1 ± 0.2). In regard to GFs, similar levels were detected in both methods. Furthermore, no morphological change or platelet activation was found following the use of the ultrasound device.

In another study, Gifford et al. [123] reported a portable system for processing whole blood without the need for centrifugation in the obtention of PRP (Figure 3(c) (A)). They took 500 mL of whole blood, using half for their passive portable system and the other half for the centrifugation process. A 1 L blood transfer bag was filled with 250 mL of blood and then placed between two plates with a compression apparatus at a slight incline of 10° to facilitate the separation of RBCs and WBCs from PRP components. After 150 min of sedimentation, the supernatant PRP was redirected to a microfluidic platelet concentrator at a flow rate of 3.2 mL min^−1^ to obtain PRP and PPP. The entire process for 250 mL took about 3 h (Figure 3(c) (B)). The results revealed that the passive system obtained 30 mL of PRP with a platelet recovery (%) of 88 ± 4, compared with the centrifugation process, which showed a platelet recovery of 96 ± 3. Nevertheless, the passive system removed more WBCs than the centrifugation method (0.4 ± 0.4 versus 5 ± 2), whereas the centrifugation method obtained higher platelet count (873 ± 146 versus 1151 ± 383). In addition, the authors proved that there was less platelet activation with the passive method than with the centrifugation-based one.

The main limitations of the alternative methods described above are their long processing time and the need to use diluents or buffers to obtain the PRP, issues that are avoided when using centrifugation. Although the ultrasonic system developed by Wu et al. [122] includes all the necessary characteristics for a commercial product, it has not yet been commercialised, and these systems are yet to replace those based on centrifugation.

### 3.2. Microscale Systems

Microscale systems are those created to obtain lower volumes of PRP (less than 1 mL). Within these systems, microfluidic devices designed for the separation of blood components have played a very important role in recent years.

Microfluidic technology enables the automated and efficient processing of liquid samples. It is one of today's fastest growing fields of research, continuously growing in economic importance worldwide [124]. The development of autonomous, portable and fully integrated microfluidic devices has the potential not only to improve process automation but also to reduce costs for industry as well as reduce economic and social stress on the healthcare system [125]. There are several microfluidic architectures that allow for the separation of plasma and blood cells and which, depending on the method of separation, are classified as active or passive systems [126, 127]. Aside from these, there are other techniques such as the compact disc (CD) microfluidic-based method to separate the plasma [128, 129]. Active technologies are based on microelectromechanical systems and use external mechanical forces for fluid control, such as dielectrophoresis or acoustophoresis [128, 130]. Passive technologies, on the other hand, do not include external forces for fluid control but rely on hydrodynamic forces, inertial forces, filtration, or sedimentation, among other techniques [131]. Several microfluidic systems have been developed for the obtention of PRP, and each of them make use of both types of technology.

**Figure 4:**
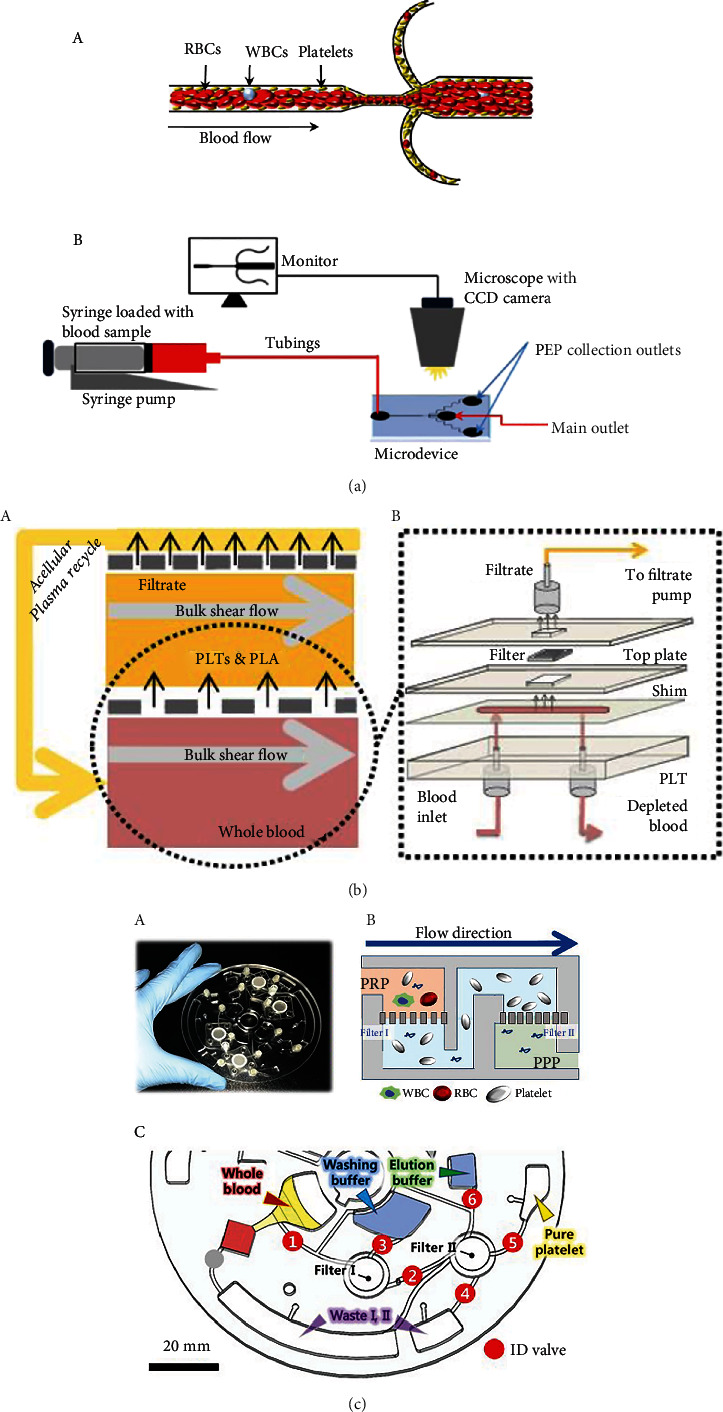
Methods to obtain small volumes of PRP. (a) Hydrodynamic system for platelet enrichment. (A) Section of the microfluidic platform. (B) Experimental set-up for platelet enrichment. (b) Microfluidic platform for the obtaining of small and high volumes of PRP. (A) Representation of the channels where the blood and plasma are passed through the filtering unit. (B) Graphical representation of the different parts of the device. (c) CD format device to obtain PRP. (A) Microfluidic CD shape platform. (B) Filtering system for the capturing of blood components and platelet collection. (C) Overview of all the components of the CD device for PRP preparation.

Similarly, Laxmi et al. [119] developed a hydrodynamic system whose mechanism of operation consisted in a platelet separation and enrichment polydimethylsiloxane (PDMS) microdevice which used a combination of biophysical effects (Figure 4(a) (B)). One of these effects—the Fahraeus effect—occurs when blood flows into the microchannel and the deformable RBCs tend to move to the centre of the channel, forming a cell-free area adjacent to the wall of the microchannel. Due to the hydrodynamic effect on the RBCs and platelets, these are pushed towards the wall and conducted towards a microchannel bifurcation where the platelets collect (Figure 4(a) (A)). The team's system achieved an 8.7-fold enrichment using undiluted whole blood with 25.5% platelet purity. The flow rate used to obtain said enrichment was one of 0.4 mL min^−1^. A platelet activation test revealed less platelet activation compared with a conventional centrifugation method. The enrichment and purity of platelets depended on the flow rates employed: at very low flow rates, the performance decreased, whereas at high flow rates, the quality of the platelets obtained deteriorated.

In addition, Dickson et al. [132] proposed a platelet separation and enrichment system using a filtration-based microdevice. This filtration system was based on crossflow filtration with two filtering steps (Figure 4(b) (A)). Blood was passed continuously through the filters for uninterrupted separation and enrichment (Figure 4(b) (B)). Due to the pore size of the first filter, RBCs were removed, whereas the second filter provided plasma with no platelets. The PRP obtained was collected, while the acellular plasma was recycled together with the rest of the blood and passed through the filtering system again in order to obtain the desired platelet concentration. Although they established a flow rate of 100 *μ*L min^−1^ at a shear rate of 16000 s^−1^ for the 30-min experiment, they believe that the study could be transformed into a macroscale device able to obtain 50 mL of platelet-enriched solution within the same amount of time.

Meanwhile, the lab-on-a-disc technique reported by Kim et al. [129] consists in a CD format device with microfluidic channels (Figure 4(c) (A)). This group proposed a novel system consisting of a tangential-flow-filtration CD format device composed of two integrated track-etched polycarbonate membranes of different pore sizes for particle filtration (Figure 4(c) (B)). The disc is composed of several channels and chambers into which less than 1 mL of whole blood is added. The disc is made to rotate, and the blood components are passed through both filters for 20 min. The first filter removes RBCs and WBCs, generating PRP by separating platelets from other cells, while the other removes the residual plasma. The last filter is responsible for concentrating the platelets in the desired volume (Figure 4(c) (C)). Compared to the centrifugation method, the plasma obtained in this way had a 4-fold platelet count with a purity of 99% and with minimum WBC contamination in the sample. Specifically, 30.3 ± 2.4 WBCs per 106 platelets were found in the plasma fraction obtained using the disc, while 10260.2 ± 6867.1 WBCs per 106 platelets were present in the sample prepared using the manual method. The device worked with nondiluted blood, but a washing buffer and an elution buffer were necessary to separate the blood components and obtain PRP.

In general, both centrifuge-based and noncentrifuge-based methods to obtain PRP focus on platelet concentration and removing the RBCs and WBCs without considering the other molecular components in the plasma. Although microscale systems have the capacity to obtain high volumes of PRP for its application in therapy, their main limitations are the high platelet fold obtained in the final formulation (ranging from four to nine), and it is not clear whether this is beneficial for treatment using PRP.

## 4. New Method to Obtain PRP Focused on the Modulation of the Molecular Composition

In addition to the platelet components, plasma also contains extraplatelet biomolecules and structures such as microvesicles and exosomes [133] with an important biological activity, which are key in processes related to cell communication and signalling [134]. Among the extraplatelet molecules, IGF-1 and HGF are the ones derived to a greater extent from the liver than from platelets [135, 136] and take part in the regulation of the chemotaxis, cellular differentiation, and mitogenesis [137]. They are also involved in the synthesis of collagen and matrix to promote the formation of fibrous connective tissue and scar formation [138]. A recent publication has shown that an increased concentration of the growth factor IGF-1 in PRP samples is associated with an increased cell viability [139]. Additionally, there is almost unanimous agreement that IGF-1 declines in serum with increasing age [140–142], being a direct relationship to the effectiveness of PRP, as at elderly ages, the effectiveness decreases. HGF is found in PRP formulations in a less concentration as it is present inside platelets but mostly as an extraplatelet molecule. This factor is important as it regulates cell growth, promotes cell migration and extracellular matrix formation, and has anti-inflammatory and antifibrotic effects [136, 143].

When applying centrifugation methods, platelets are concentrated, but not extraplatelet biomolecules. This occurs because the centrifugation method does not work at the molecular level. In fact, obtaining these molecules involves the use of very high-speed centrifugations, called ultracentrifugations [144], that are not compatible with cell viability as platelets are activated [145] and precipitated [146] during the process.

**Figure 5:**
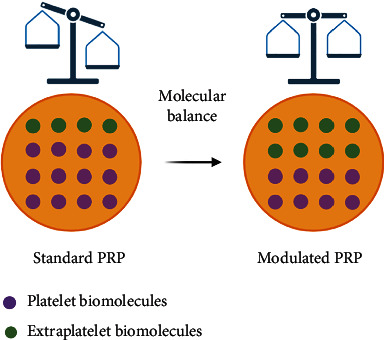
Representation of the biomolecule balance for the obtaining of modulated PRP. In standard PRPs, biomolecule imbalance is generated after platelet activation resulting in higher levels of platelet-derived growth factors than extraplatelet ones. Balancing the concentration of those molecules could potentially enhance the effect of the treatment.

Thus, the efficacy of PRP can be optimized by modulating the PRP molecular cocktail, affecting both platelet and plasma biomolecules. The regulation of the molecular balance (Figure 5) between the different PRP biomolecules could favor certain biological processes [147]. It is known that tissue repair is provided by the physiologically natural balance/ratio of GFs and other cytokines, which contain anabolic and catabolic functions in supraphysiologic concentrations, directly into the site of injury optimizing the healing environment [51, 148, 149]. Preserving a natural ratio of these biomolecules may allow the maintaining of the body's homeostatic environment, which theoretically would provide an abundance of healing factors without disrupting in vivo cellular functions [69, 147].

### 4.1. Methods to Concentrate the Molecular Components of PRP

#### 4.1.1. Modulation of the Cytokine Content

In recent years, in an attempt to achieve an improvement in PRP treatments, different PRP formulations have been developed by varying the ratio between certain biomolecules [147].

**Figure 6:**
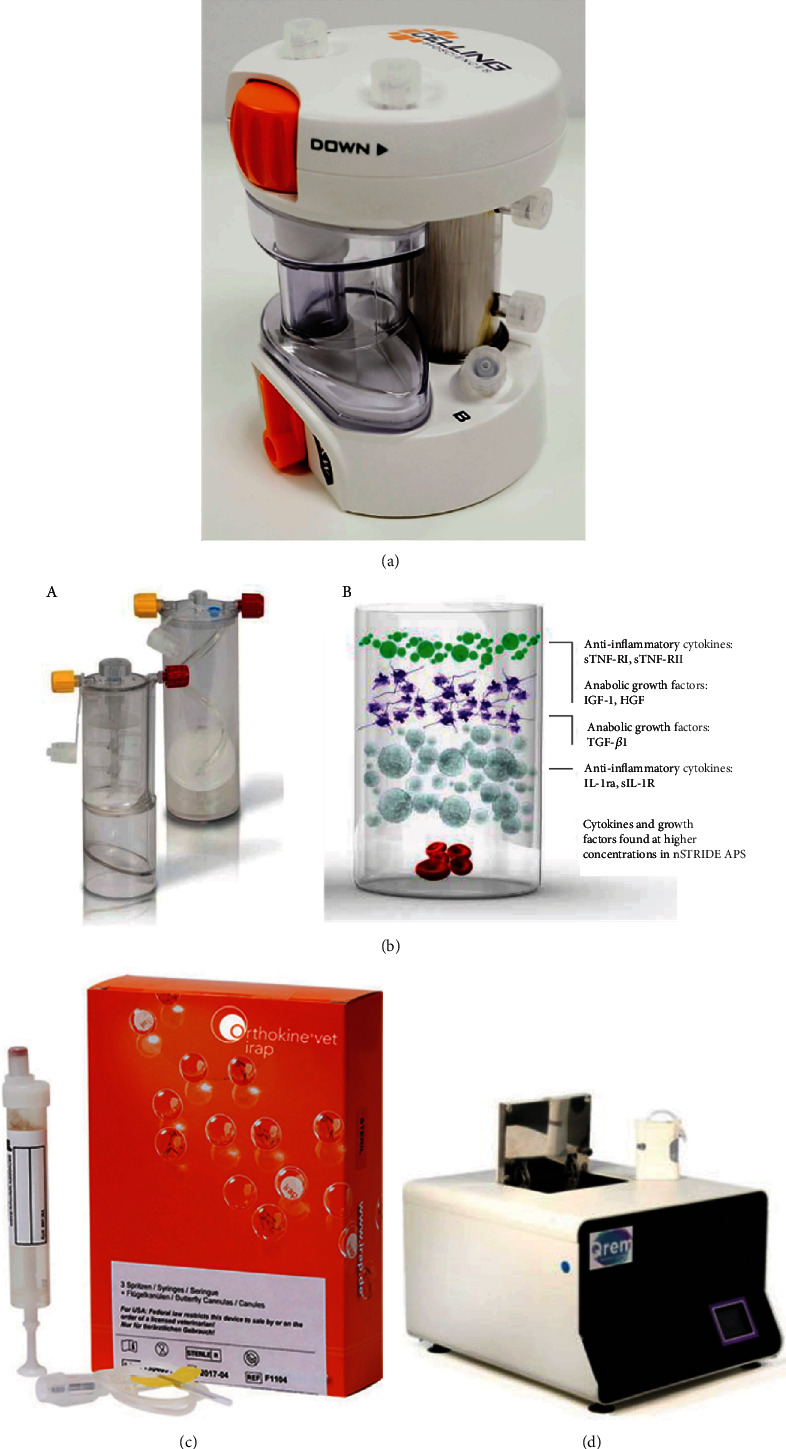
Devices to obtain novel formulations of PRP. (a) Plasma ultrafiltration system for PRP obtention. (b) Plasma concentration system by the use of acrylamide hydrogel beads. (A) Devices used for the separation of blood components and the final concentration of plasma with acrylamide beads. (B) Bioactive molecules present in the final plasma. (c) Orthokine protein-based autologous solution. (d) Qrem protein-based autologous solution: both the device and the kit for the obtention of the protein-based solution are shown.

The first is that launched by the company Celling Bioscience, called the ART PRP Plus kit [150], which has an integrated filter system of nanoporous fibres for the ultrafiltration of proteins from the PRP (Figure 6(a)). This system removes RBCs and WBCs by the use of centrifugation, enriches platelets, and, in turn, concentrates extraplatelet biomolecules of a molecular size greater than 25 KDa by means of the nanoporous filter. From among all the proteins present in the plasma, this system concentrates the proteins VEGF, PDGF, TGF-*β*, FGF, alpha-2 macroglobulin, interleukin-1 receptor antagonist protein, and fibrinogen. Extraplatelet IGF-1 factor is not concentrated due to its molecular weight of 7.65 KDa [135].

The second is the protein-based solution provided by Zimmer Biomet nStride®, an Autologous Protein Solution Kit, which is used for the concentration of extraplatelet molecules (Figure 6(b) (A)). This kit consists of a cellular concentration system that concentrates anti-inflammatory cytokines and anabolic GFs to significantly reduce pain and promote cartilage health. It generates a type of PRP that, in the last stage of its production, concentrates all the proteins using polyacrylamide beads resulting from the absorption of the water from the plasma [151, 152]. However, this system is based on obtaining PRP that contains both leukocytes and erythrocytes, and although leukocyte content is controversial in the effectiveness of its use in PRP therapies, erythrocytes can be detrimental, for example, to joint structures [153] (Figure 6(b) (B)). Moreover, the concentration of extraplatelet biomolecules in this system occurs after platelet activation, so a balance between platelet and plasmatic factors is not achieved.

Another new therapeutic, protein-based formulation is used in Orthokine autologous therapy (Figure 6(c)). This treatment is based on the production of anti-inflammatory cytokines such us IL-4, IL-10, and IL-1Ra, which is an antagonist of the proinflammatory cytokine IL-1*β*. The technique involves incubating whole blood from the patient in a syringe that contains CrSO4 surface-treated glass spheres, for 24 h at 37°C in an incubator. The contact of whole blood with these beads leads to a rapid increase in the concentration, which can be up to 140-fold in the case of the IL-1*β*Ra, as CrSO4 initiates the activation of monocytes and the release of pro- and anti-inflammatory cytokines [154]. After 24 h of incubation, blood is centrifuged to collect the plasma fraction [155].

Last but not least, Qrem company has launched a new protein solution based on Orthokine and nStride kits (Figure 6(d)). The Qrem cytokine kit [156] is composed of two containers, one of which is filled with 18 mL of the patient's whole blood, while the other is filled with 20 mL. The kit is placed on a special device which automatically performs all the processing steps to obtain the final autologous solution. The first steps in the process yield a platelet and leukocyte concentrate in one of the containers while, in the other, the patients' serum is obtained. This primary serum contains autologous activating elements such as thrombin and calcium, as well as anti-inflammatory cytokines and GFs. At the end of the process, the contents of both containers are mixed, which leads to the activation of the platelets and leukocytes, and the formation of a fibrin matrix.

#### 4.1.2. Modulation of Growth Factor Content

Many novel PRP-obtaining methods focus on the modulation of pro- and anti-inflammatory cytokines for the improvement of the effectiveness of the therapy. However, none of these systems focus on the concentration of certain GFs. A significant factor in the regenerative effect of PRP is due to the different growth factor content. By concentrating platelets and activating them, platelet-derived factors are released into the plasma medium; however, extraplatelet factors such as HGF and IGF-1 (present at higher concentrations in circulating plasma) are maintained at the same basal levels as in blood. In addition, it has been shown that a higher concentration of some factors and a decrease in others can cancel out the effect of certain GFs. Therefore, the modulation of these factors could be key to the improvement of PRP-based treatments [147]. Based on that GF concentration, several methods have been developed where part of the water content from the plasma is removed for the concentration of platelets and GFs.

Several methods could be used to obtain those new PRP formulations, some of them involving microfluidics. For example, a system was developed in which a plasma fraction previously obtained by centrifugation is circulated through a system based on microfluidics [157]. This system generated the evaporation of water from the plasma, dehydrating it and concentrating both platelets and extraplatelet plasma molecules. At the same time, the sample runs to another microfluidic platform in which the concentrated electrolytes were dialyzed. However, this system was designed for very small volumes of PRP, and its transfer to volumes applicable to clinical practice made it unfeasible due to the excessive time involved for sample preparation and to coagulation problems during the evaporation process.

**Figure 7:**
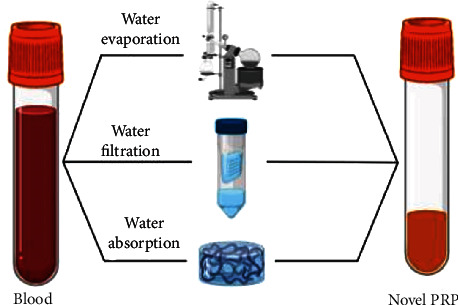
Novel methods for the obtaining of a PRP formulation enriched in platelet and extraplatelet growth factors. Three different methods were developed by evaporating, filtrating, and absorbing the water content from the plasma to double the amount of platelet content and extraplatelet factors.

Mercader Ruiz et al. developed three different methods [156, 158, 159] (Figure 7) for the obtaining of a novel PRP enriched not only in platelets but also in extraplatelet growth factors by means of removing part of the water from the plasma. The evaporation method removed the water content from the plasma by means of a rotary evaporator at a controlled temperature of 37°C for the obtaining of a novel PRP with an increase in platelet and protein levels. The enrichment increases the extraplatelet IGF-1 levels, inducing higher proliferation when the novel PRP was tested in human dermal fibroblasts at 96 h compared to a standard PRP, which presents only platelet enrichment and similar levels of proteins compared to basal blood levels.

The filtration method focused on the removal of the water content from the plasma to eliminate unwanted components such as RBCs and WBCs and concentrate platelets and proteins after their subsequent dilution in a PRP fraction, obtaining a novel PRP with higher levels of platelets and extraplatelet growth factors. This method enables the concentration of the extraplatelet GFs HGF and IGF-1, obtaining higher cell proliferation capacity of human dermal fibroblast at 96 h compared to the standard PRP.

The last method focuses on the obtaining of a PRP formulation by means of an antifouling hydrogel-based technique. This method enables the concentration of the extraplatelet HGF and IGF-1 by putting in contact a hydroxyethyl acrylamide-based hydrogel developed by Zhao et al. [160] at a concentration of 0.1 g mL^−1^ during 5 min in a blood plasma fraction. The resulting novel PRP presents higher cell proliferation capacity when cultured in human dermal fibroblast for 96 h comparing to a standard PRP.

## 5. Conclusions

PRP is an autologous biological product used in the field of regenerative medicine due to its great capacity to regenerate tissue, its easy preparation, and its low-cost procedure.

Centrifugation is mainly used to obtain a plasma fraction from whole blood with different concentrations of both platelets and WBCs. Depending on the treatment to be applied, one formulation or another may be produced, applying different centrifugation programs in which the force, temperature, and time can vary. There is no consensus on the ideal formulation of PRP regarding concentration of platelets to ensure a therapeutic effect, although experts recommend 1.5–3 times to the basal level of platelets in whole blood.

In order to minimize sample manipulation, reduce processing times, and avoid platelet activation, novel methods beyond centrifugation have been developed. A major disadvantage of these noncentrifugation-based methods, similarly to the centrifugation-based methods, is that they focus on increasing the concentration of platelets, neglecting the effect of an unbalanced ratio between platelets and soluble extraplatelet factors. This is very relevant because blood plasma contains many biomolecules whose combination can have a synergistic effect potentially making treatments with PRP more effective. Therefore, along with platelets, it might be recommended to concentrate plasma biomolecules in order to keep the homeostatic ratio between platelets and extraplatelet biomolecules in PRP, as depending on the cell type, the pathology, or even the chronicity of the injury, it can be beneficial for tissue repair.

New methods have been developed over time to produce PRP formulations with increased concentration of platelets and soluble factors, but often, the new formulations contain an increased concentration of only selected biomolecules leaving others aside. In fact, the growth factors IGF-1 and HGF are present at high concentration in the extraplatelet environment and are not usually concentrated in conventional PRPs. Some have even kept RBCs and WBCs in their formulations, not being beneficial in many cases.

In short, this review gives an overview of novel systems for PRP production. Considering that many devices have focused on the concentration of certain proteins of the plasma without regard to achieving a balance between extraplatelet and platelet biomolecules of the PRP, this review emphasizes the need to investigate new formulations of PRP that can improve the efficacy of the treatment.

## Figures and Tables

**Table 1:** Classification of the main biological therapies.

**Biological therapy**	**Action**
Hemoderivative products (PRP and others)	Promote tissue regeneration and reparation for use in applications ranging from the musculoskeletal system to aesthetics and oral medicine [5].
Vaccines	Exploit human immune system to respond to, and remember, encounters with pathogen antigens and protect from catching a disease [6].
Antitoxins	Neutralize unbound exotoxins to eliminate the toxin from the body [7].
Recombinant proteins (insulin or erythropoietin)	Use of recombinant hormones, interferons, interleukins, growth factors, blood clotting factors, or enzymes, among others for the treatment of major diseases such as diabetes, multiple sclerosis, thrombocytopenia, hepatitis, and asthma [8].
Recombinant nucleic acids	Inhibit DNA or RNA expression, thereby halting the production of abnormal proteins related to a disease while leaving all other proteins unaffected [9].
Interleukins	Regulate immune responses. They are used as biological response modifiers to boost the immune system in cancer therapy [10].
Monoclonal antibodies	Use in the diagnosis and treatment of many diseases, including some types of cancers. They can be used alone or carrying drugs, toxins, or radioactive substances directly to cancer cells [11].
Stem cell therapies	They are a form of regenerative medicine designed to repair damaged cells within the body by reducing inflammation and modulating the immune system [12].
Gene therapies	Replacement of a faulty gene or add/silence genes in an attempt to cure or help fighting a disease. They are used for treating cancer, heart disease, diabetes, hemophilia, or AIDS [13].
Exosomes	Exosomes exhibit disparate functional and phenotypic characteristics. They transfer biological messages from donor cell to recipient cell, which makes exosomes as a novel therapeutic platform delivering therapeutic molecules to the target tissue or cells [14].
Cell secretome therapies	Cell secretome therapy relies on the delivery of cell content, either autologous or allogeneic, to promote tissue repair and regeneration. These secretory factors play important roles in many biological functions, including homeostasis, apoptosis, proteolysis, cytokines, and inflammation, among others [15].

**Table 2:** Biological functions in tissue regeneration of each GF.

**Growth factor**	**Abbreviation**	**Biological function**
Insulin-like growth factor	IGF-1	Predominates in plasma, produced by the liver. Promotes cell growth, proliferation, and differentiation.
Transforming growth factor-*β*1	TGF-*β*1	Acts in the early cellular reparative response (migration of cells and angiogenesis) in the wound area. Enhances synthesis of collagen and inhibits osteoclast formation and bone resorption.
Platelet-derived growth factor	PDGF	Enhances collagen synthesis; promotes mitosis and chemotaxis of mesenchymal origin cells, proliferation of bone cells, and macrophage activation; and stimulates vasculogenesis and angiogenesis.
Hepatocyte growth factor	HGF	Regulates cell growth, migration, and morphogenesis. Promotes extracellular matrix synthesis and has anti-inflammatory and antifibrotic effects.
Vascular endothelial growth factor	VEGF	Is a key mediator in wound healing and the main inducer of angiogenesis due to the stimulation of chemotaxis and proliferation of endothelial cells. Also, it stimulates chemotaxis of macrophages and neutrophils.
Fibroblast growth factor	FGF	Is a potent inductor of cell proliferation, angiogenesis, and differentiation. Stimulates the growth and differentiation of chondrocytes and osteoblasts. Inhibits osteoclastic actions.
Epidermal growth factor	EGF	Promotes chemotaxis, mitogenesis, and cytokine secretion by epithelial and mesenchymal cells.
